# Transcriptome Dataset of Halophyte Beach Morning Glory, a Close Wild Relative of Sweet Potato

**DOI:** 10.3389/fpls.2016.01267

**Published:** 2016-08-24

**Authors:** Robert W. Reid, Yan Luo, Sue Yan, Thomas E. Miller, Bao-Hua Song

**Affiliations:** ^1^Department of Bioinformatics and Genomics, University of North Carolina at CharlotteCharlotte, NC, USA; ^2^Department of Biological Sciences, University of North Carolina at CharlotteCharlotte, NC, USA; ^3^Key Laboratory of Tropical Forest Ecology, Xishuangbanna Tropical Botanical Garden, Chinese Academy of SciencesMenglun, China; ^4^Miller Lab, Department of Biological Science, Florida State UniversityTallahassee, FL, USA

**Keywords:** sweet potato, morning glory, salt tolerance and sensitivity, RNA-sequencing, halophyte

## Introduction

Soil salinity is one of the major environmental factors causing crop loss worldwide. Currently, 33% of the global arable land is affected by salinity, hampering crop production in these fields (Flowers and Colmer, 2008). As the global population continues to rise, crop production is facing increasing demands (Flowers and Muscolo, 2015). With the combined pressures to sustain or even increase the world's food supply, salt tolerance is becoming an important agronomic trait to support crop plant growth and production in marginal and high saline soils.

Salt tolerance is a genetically complex trait that has evolved independently by different mechanisms in numerous lineages (Bromham, 2015). Efforts to improve salt tolerance in crops through selective breeding have proven difficult (Ruan et al., 2010) due to a lack of genetic resources and limited salt tolerance associated with known molecular markers (Deinlein et al., 2014).

We conducted a transcriptome analysis of a wild relative of the salt-sensitive sweet potato (*Ipomoea batatas*): the beach morning glory (*Ipomoea imperati*). Beach morning glory is a halophyte that thrives in beach ecosystems of high salt content. Our objective was to better understand the genetic basis for salt tolerance in *I. imperati*, so that future studies might transfer the salt tolerance genes into sweet potatoes.

## Value of the data

- Beach Morning is closely related to sweet potatoes, but commonly grows in high salt conditions. This creates a potential genetic source for adding much-needed salt tolerance into future sweet potato breeding strategies.
- To date, there is no well-characterized transcriptome for either sweet potato or morning glory and no source for gene annotation when exposed to high levels of salt. This dataset of biological triplicates can help in the further understanding of the plant pathways involved under varying salt levels.
- These data will help identify relevant genes that are significant differentially expressed under salt stress as well as identify genes that are detectable under normal growing conditions in both root and leaf tissue. Gene expression can be compared between the 2 tissue types to identify how different tissues respond within the plant to salt exposure.

## Data

### Experimental design, materials, and methods

#### Plant materials

##### Total RNA extraction and quality control, library preparation, and RNA-seq

Seeds from *I. imperati* were collected from St. George Island, Florida as seed and grown in the lab. At 2 weeks of growth 600 mM NaCl solution (treatment) or water (control) was applied to the soil, daily, for 7 days. Three biological replicates for each treatment and species were harvested at 0, 3, 24 h, and 7 days. Total RNA was extracted from the roots and leaves using the Qiagen protocol and treated with DNAse (Qiagen). Quantity and integrity of the extracted total RNA were determined using an Agilent 2100 bioanalyzer (Agilent), respectively, to be RIN >9. A total of 12 RNA-Seq libraries, including three biological replicates, were prepared using Illumina TruSeqRNA sample Preparation Kit (Illumina). Twelve normalized cDNA libraries were constructed and sequenced using the Illumina Hiseq2500 platform (North Carolina State University) to generate 100 bp paired-end raw reads.

Raw reads were deposited into the Short Read Archive (SRA database, http://www.ncbi.nlm.nih.gov/sra) with the following accession information:

- Bioproject ID = PRJNA322032
- Biosample accession
  - Roots = SAMN05007696, SAMN05271550, SAMN05271551
  - Leafs = SAMN05271552, SAMN05271553, SAMN05271554
- SRA Root tissue experiment = SRX1771615, SRX1858743, SRX1858745
- SRA Leaf tissue experiment = SRX1858747, SRX1858786, SRX1858810

Root and leaf tissue experiments contain sequence reads of triplicate runs for both salt treated and control.

##### Transcriptome *De novo* assembly

Sequence reads were filtered using the Fastx-toolkit (Gordon and Hannon, 2010) for quality and adapter removal using the fastq_quality_trimmer tool with the following parameters: -Q33 -v -t 20. Paired ends were corrected and repaired using Perl script, PE_FIX_POSTQC.pl (all scripts described herein are available at https://github.com/bioinformagical/SweetPotatoRNA-Seq). Paired reads were validated using validateHiseqPairs.pl.

Reads were combined across all conditions and *de novo* assembled via Trinity (Grabherr et al., 2011; version r2013-02-25) using default settings in order to build a suitable set of reference contigs (column 4 of Table 1). These contigs are used for the purposes of determining differential gene expression and pathway level analysis (paper in preparation). Assembly is publicly available on Figshare at: https://figshare.com/articles/Morning_Glory_Transcriptome_assembly/3498239.

**Table 1:** **Summary of assembly, from sequencing reads produced to final unigenes assembled**.

**Pre assembly**	**Number of reads**		**Post assembly**	**Number of sequences**
Raw reads	Leaf + Root	252,166,154	Trinity	94,728
Filtered reads	Leaf + Root	201,357,272	CD-Hit “cluster sequences”	67,911
Salt treated	Leaf x3	29,577,252	Transdecoder	50,668
condition	Root x3	23,409,894	proteins	
Control	Leaf x3	17,581,336	Matches to	39,902
condition	Root x3	27,194,620	NCBI NR	

There is roughly 10X coverage for the transcriptome assembly.

Trinity contigs of high similarity were clustered into groups with CD-HIT-EST (version v4.6.1-2012-08-27) and a single representative from each cluster was used as a reference sequence for read alignment. Clustered contigs are publicly available on Figshare at: https://figshare.com/articles/CDHIT_Cluster_of_assemblies/3498263.

##### Predicted proteins and initial annotation

Sequences from each CD-HIT cluster were transdecoded into predicted proteins using Transdecoder (http://transdecoder.github.io), a software tool that identifies the most likely protein sequence by finding the longest open reading frame and comparing the translated protein sequence to known proteins in the PFAM domain (Haas et al., 2013). Only proteins greater than 100 amino acids in length were retained for further annotating. After protein translation, 50,688 predicted proteins were found (column 4 of Table 1). Sequences are available on Figshare at: https://figshare.com/articles/Morning_Glory_Predicted_proteins/3498308.

Sequences were initially annotated by blasting nucleotide sequences against the NCBI NR database (BLASTX, -evalue 1e-10 -soft_masking true -max_target_seqs 1) where 39,902 sequences had a match. The most commonly occurring matches were to genes from *Solanum lycopersium*. We mapped the CD-HIT clusters onto the records from the GO database and retrieved 21,418 GO annotations. BLAST2GO assigned 18,034 with terms of “Biological process,” 13,322 with terms of “cellular component,” and 17,134 with terms of “molecular functions.”

## Author contributions

BS: conceived the idea and acquired funding; YL, BS, SY, TM: collected seeds and conducted the experiment; RR: performed analysis on the data; RR, YL, BS, SY, TM: wrote the manuscript.

### Conflict of interest statement

The authors declare that the research was conducted in the absence of any commercial or financial relationships that could be construed as a potential conflict of interest.
